# Climate and fishing steer ecosystem regeneration to uncertain economic futures

**DOI:** 10.1098/rspb.2014.2809

**Published:** 2015-03-22

**Authors:** Thorsten Blenckner, Marcos Llope, Christian Möllmann, Rudi Voss, Martin F. Quaas, Michele Casini, Martin Lindegren, Carl Folke, Nils Chr. Stenseth

**Affiliations:** 1 Stockholm Resilience Centre, Stockholm University, Kräftriket 2B, Stockholm 106 91, Sweden; 2 Instituto Español de Oceanografía, Centro Oceanográfico de Cádiz, Puerto Pesquero, Muelle de Levante, Cadiz 11006, Spain; 3 Institute of Hydrobiology and Fisheries Sciences, Center for Earth System Research and Sustainability, University of Hamburg, Grosse Elbstrasse 133, Hamburg 22767, Germany; 4 Department of Economics, Christian Albrechts Universität zu Kiel, Olshausenstraße 40, Kiel 24118, Germany; 5 Swedish University of Agricultural Sciences, Department of Aquatic Resources, Institute of Marine Research, Turistgatan 5, Lysekil 45330, Sweden; 6 Centre for Ocean Life, National Institute of Aquatic Resources, Technical University of Denmark, Charlottenlund Castle, Charlottenlund 2920, Denmark; 7 Beijer Institute of Ecological Economics, Royal Swedish Academy of Sciences, PO Box 50005, Stockholm 104 05, Sweden; 8 Centre for Ecological and Evolutionary Synthesis (CEES), Department of Biosciences, University of Oslo, PO Box 1066 Blindern, Oslo 0316, Norway

**Keywords:** Baltic Sea, cod, food-web dynamics, regime shifts, shifting baseline, ecosystem-based management

## Abstract

Overfishing of large predatory fish populations has resulted in lasting restructurings of entire marine food webs worldwide, with serious socio-economic consequences. Fortunately, some degraded ecosystems show signs of recovery. A key challenge for ecosystem management is to anticipate the degree to which recovery is possible. By applying a statistical food-web model, using the Baltic Sea as a case study, we show that under current temperature and salinity conditions, complete recovery of this heavily altered ecosystem will be impossible. Instead, the ecosystem regenerates towards a new ecological baseline. This new baseline is characterized by lower and more variable biomass of cod, the commercially most important fish stock in the Baltic Sea, even under very low exploitation pressure. Furthermore, a socio-economic assessment shows that this signal is amplified at the level of societal costs, owing to increased uncertainty in biomass and reduced consumer surplus. Specifically, the combined economic losses amount to approximately 120 million € per year, which equals half of today's maximum economic yield for the Baltic cod fishery. Our analyses suggest that shifts in ecological and economic baselines can lead to higher economic uncertainty and costs for exploited ecosystems, in particular, under climate change.

## 1. Introduction

Management of depleted fish stocks has traditionally been treated as a single species concern, primarily related to the level of exploitation [1]. Understanding the dynamics of commercially exploited fish stocks in an ecosystem context, including the interactions among ecosystem components and how these components are affected by both anthropogenic and natural drivers, remains a considerable challenge [2]. This understanding is required to evaluate the chances of restoration of the target stocks and the ecological and socio-economic implications this may have [2–4]. There are different levels of recovery depending on the magnitude and duration of the perturbation [2] as well as on the focal species or group of species. Furthermore, ecosystems are inherently dynamic in the sense that they continuously develop owing to natural and anthropogenic processes. This means that even if certain population(s) recover after a perturbation, the configuration and dynamics of the ecosystem as a whole has been altered, i.e. populations may recover, but ecosystems regenerate in the face of change [5]. Natural as well as anthropogenic processes influence the restoration target or baseline [6,7]. It is therefore important to incorporate and try to account for the true nature of these interactions when evaluating management strategies.

Climate, in particular, can greatly influence ecosystem dynamics [8,9], and the compounded effects of climate and anthropogenic drivers, such as eutrophication and overexploitation, can lead to nonlinear and threshold-like responses (regime shifts) to drivers [10]. Regime shifts are sudden, persistent reorganizations in the structure and function of ecosystems [10,11], usually driven by a multitude of drivers, e.g. climate and overfishing [12]. Feedback mechanisms have been suggested as regime stabilizers that once established make the ecosystem state difficult to reverse [13]. Regime shifts have been documented in several marine ecosystems, e.g. the Black Sea [14], Mediterranean Sea [15] and North Pacific [16].

**Figure 1.**
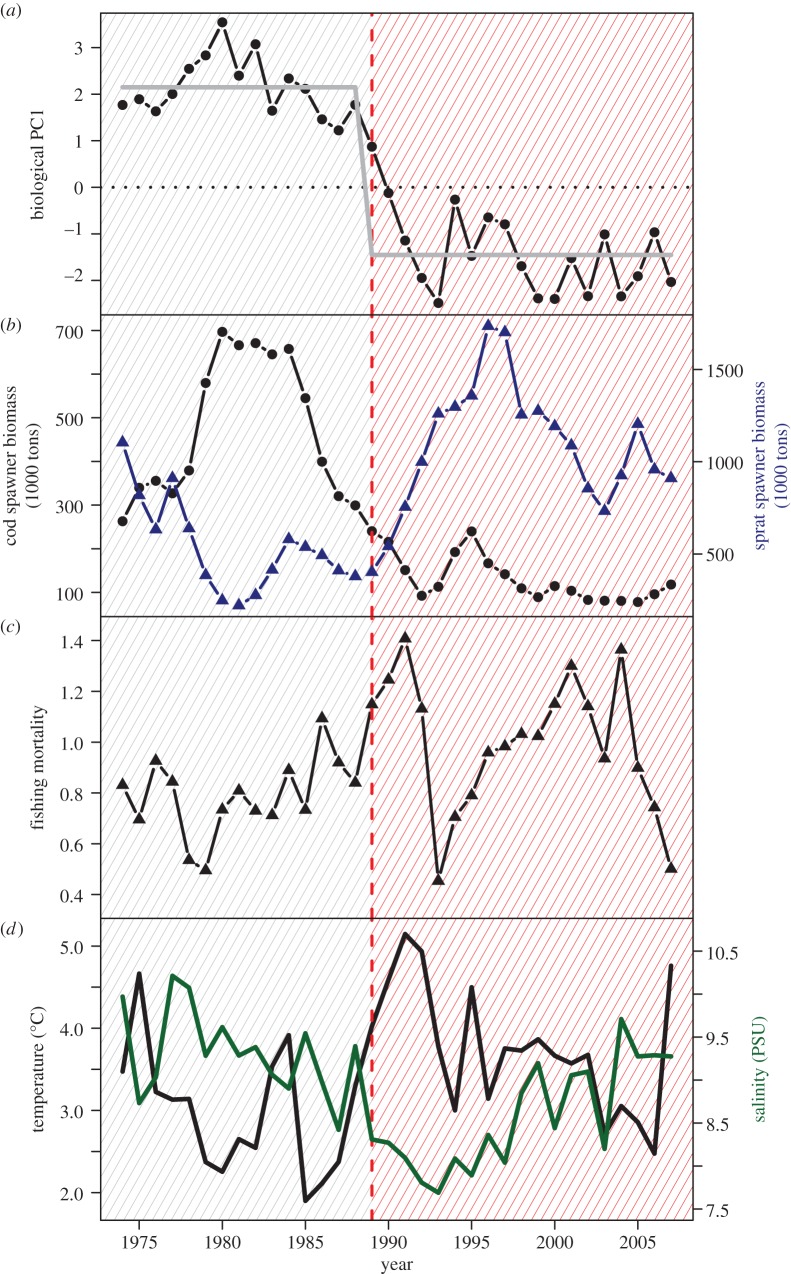
Regime changes in the Baltic Sea ecosystem. Demarcation between regimes is indicated by red dotted line and coloured background for past (grey) and current (red) regimes. Changing ecosystem structure based on the leading mode (PC1) of biotic data spanning three trophic levels and regime indicator (grey) (*a*), changes in the fish community from cod (black circles) to sprat (blue triangles) (*b*), exploitation history of cod given in terms of fishing mortality (*c*), and changes in hydroclimatic regime indicated by spring temperature (black) and salinity (green) (*d*).

In the Central Baltic Sea, a fishery- and climate-induced regime shift in the late 1980s changed the food web from being dominated by the large piscivorous cod (*Gadus morhua*) to an alternative configuration dominated by planktivorous fishes (figure 1) [17,18]. After the implementation of a multi-annual management plan [19], the cod population has shown signs of recovery [20], but the underlying causes of the increase remain controversial [21,22].

Owing to extensive data availability, several studies on ecosystem dynamics have been conducted in the Central Baltic Sea, comprising empirical analyses (e.g. [17,18,23]) as well as food-web modelling [24–26]. However, none of these studies has explicitly assessed the potential for ecosystem regeneration and its associated economic consequences under different climate conditions. Here, by focusing on cod as most important economic and ecological indicator, we evaluate the chances of the Baltic Sea to regenerate to its previous state both from an ecological and socio-economic perspective.

To do so, we develop a statistical model based on historical records over the last three decades. Our analysis incorporates direct and indirect responses to the key drivers of fishing mortality and environmental conditions (temperature and salinity) and at the same time allows for changes in these interactions depending on the configuration of the ecosystem at a time. These regime-dependent effects are accounted for by means of a modified generalized additive model (GAM) that allows the type and form of the interactions to change depending on a threshold value [27].

This modelling approach is a novel way to explore the regeneration potential of a deeply altered ecosystem by specifically incorporating feedbacks and thresholds in relation to the confounding effects of climate and fishing. The biological output of the model is then measured in terms of economic profit, consumer surplus (CS) and annual risk premium (RP) of the cod fishery. This allows us to translate the ecological regeneration potential into societal costs.

## 2. Material and methods

### (a) Data

We collected environmental and biological monitoring data representative of the dynamics of the Central Baltic Sea over the time period 1974–2011 [23,28] (electronic supplementary material, table S1). In this area, the three commercially and ecologically most important fish stocks are cod (*G. morhua*), sprat (*Sprattus sprattus*) and herring (*Clupea harengus*) [29]. The mean annual fishing mortality (*F*) for each species was used to represent the exploitation pressure exerted on them by the commercial fishery [30]. The dominant zooplankton taxa were characterized by spring (May) and summer (June–August) biomass of the copepods *Pseudocalanus acuspes*, *Acartia* spp. and *Temora longicornis,* as well as summer biomass of cladocerans [31]. Chlorophyll *a* from both spring and summer was included as a proxy for phytoplankton biomass. The biological data differ in their spatial dimension (electronic supplementary material, table S1). The annual fish stocks are generally assessed for areas encompassing their geographical distribution. In our dataset, cod and herring are representative for the Central Baltic Sea, while the sprat stock is assessed for the whole Baltic Sea [30]. The zooplankton data were sampled in the Gotland Basin, a sub-basin of the Central Baltic Sea [18], but temporal trends are largely representative for the entire Central Baltic Sea [32]. Chlorophyll *a* from both spring and summer were used from the Gotland Basin.

The abiotic conditions were represented by sea surface temperature in spring (May) and summer (July), mid-water temperature (40–60 m) in spring and summer, and mid-water salinity (80–100 m) in spring, all sampled in the Gotland Basin. In addition, the annual cod reproductive volume for the whole Central Baltic Sea, i.e. the volume of water with appropriate salinity (above 11 PSU) and oxygen (more than 2 mg l^−1^) conditions for cod egg survival [33], as well as the Baltic Sea Index, a regional atmospheric pressure index reflecting the effect of climate variability on oceanographic processes in the Central Baltic Sea area [34], were included as explanatory variables (electronic supplementary material, table S1). Note that not all these variables were finally retained (see Model selection section).

Regime shift detection in real ecosystems is challenging and a number of methods have been proposed, e.g. [35,36]. We applied a principal component analysis to the observed biological data, which includes cod, sprat, herring, *P. acuspes* and cladocerans*.* The first principal component (PC1) of this data subset was used as an indicator for the ecosystem state as we expected to find a change across all trophic levels. A sequential *t*-test with a *p* < 0.05 and a cut-off length of 10 years was subsequently performed on this proxy [35] and a significant break was detected in 1989 (figure 1*a*). This step-wise change supports the hypothesis of the existence of two distinct regimes in the biological configuration of this ecosystem [18].

### (b) Statistical modelling, a four-step approach

Our modelling approach comprised four steps: (i) fitting separate statistical models for each trophic level; (ii) coupling the individual models into a ‘joint food web model’ that reproduces observed population dynamics based on external drivers and the trophic interactions emerging from the individual models [36]; (iii) exploring the regeneration potential of the food web in response to decreasing exploitation rates under past and current temperature and salinity conditions; and finally (iv) assessing the economic consequences for the commercial cod fishery (§2c).

#### (i) General model set-up and individual model selection

**Figure 2.**
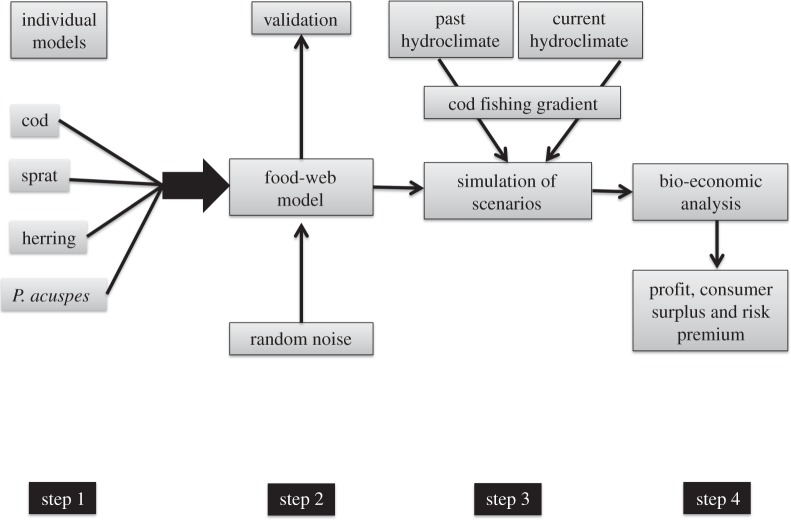
Study design to test for ecosystem regeneration pathways. The schematic describes the four steps used in our study.

To be able to account for linear, nonlinear, as well as regime-dependent relationships, we used two types of GAMs [37,38] (figure 2, step 1): (i) fully additive or common GAM, which assumes that the effect of each covariate is stationary, i.e. that the form of the relationship does not change over time; and (ii) non-additive threshold GAM (tGAM), which, contrary to the former, allows the type of relationship between the response and explanatory variables to change below and above a certain value of a threshold variable. The threshold is estimated from the data and chosen by minimizing the generalized cross-validation (GCV) criterion [38]. We selected cod biomass as the threshold variable as the biomass of this top predator has been shown to control the food-web dynamics in the Central Baltic Sea [18].

Individual model selection was based on a stepwise approach, aimed at excluding covariates with a *p*-value > 0.05 and minimizing the GCV criterion of the model [27]. The underlying statistical assumptions of all models, whether GAMs or tGAMs were tested (see details in the electronic supplementary material). To avoid over-smoothing, which is likely to occur with small datasets, we let the effective degrees of freedom (edf) be restricted to a maximum of four for GAMs and three when using tGAMs. Also, for tGAMs we used only one intercept over the whole range of conditions and not one per regime (see equation (2.2) in the electronic supplementary material). By doing so, we ensure that the average level of the response variable for a given regime, whether lower or higher than in the alternative regime, is simply the result of the additive effect of the various environmental covariates and trophic interactions described by the model for that regime. Allowing one intercept per regime would have increased the explanatory variance but at the same time would have reduced the parsimony of the model (one more parameter) and, more importantly, possibly mask other potential relationships. The same applies if we had used any temporal information, e.g. separating the dynamics before/after the threshold year. This is important as we aimed at simulating the ecosystem over a range of conditions without having to use regime (or time) as an explanatory variable.

**Figure 3.**
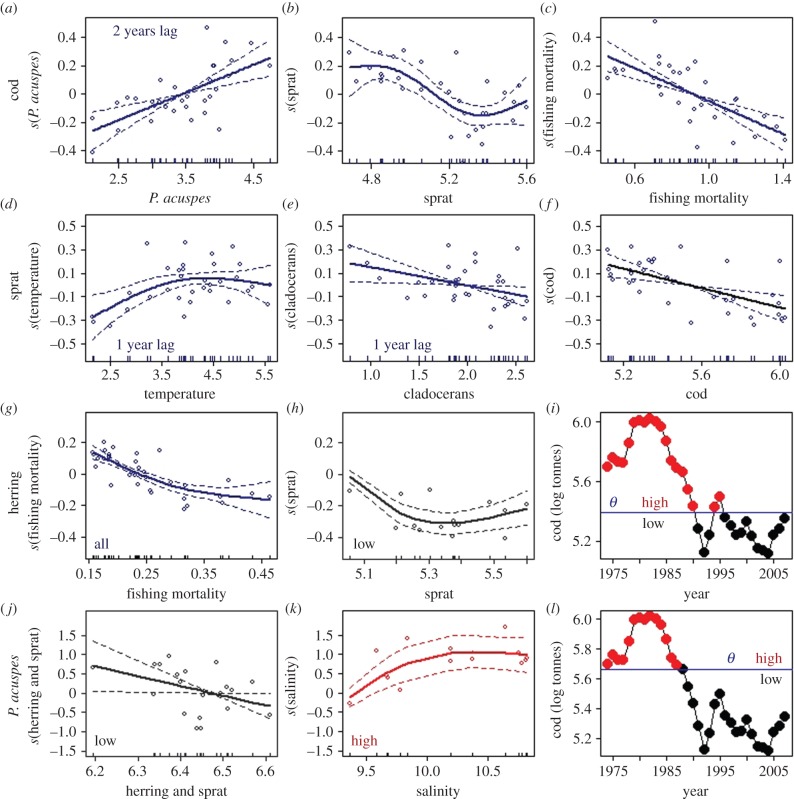
Individual trophic-level statistical models. Each row displays partial plots showing the main biotic and abiotic effects on cod (*a–c*), sprat (*d–f*), herring (*g–i*) and *Pseudocalanus acuspes* (*j–l*). Partial plots based on models without threshold effects are shown in dark blue, while non-additive interactions above and below thresholds (*i,j*) are shown in red and black, respectively. Associated thresholds (*θ*) are indicated by horizontal lines (*i,l*). For sprat the sea surface temperature in summer (*d*) and for *P. acuspes* the spring mid-water salinity (*k*) are shown.

For *P. acuspes*, the additive formulation outperformed its non-additive counterpart. However, the residuals of the former model violated the normal distribution assumption, which affects the significance (*p*-values) of the covariates' effect. The alternative tGAM formulation met the normality assumption, agreed to previous results [17], and was therefore preferred over its fully additive counterpart. For herring, the tGAM formulation turned out to be more parsimonious than the simple GAM and was therefore retained. In total, we selected two additive and two non-additive models (figure 3; electronic supplementary material, tables S2–S5).

#### (ii) Food-web model and validation

In the next step (figure 2, step 2), the selected individual models (based on data from 1974 to 2007) were dynamically coupled into a joint food-web model, where the internal dynamics (i.e. trophic interactions) are driven solely by the external covariates (i.e. temperature, salinity and fishing) that were retained during model fitting [39]. Since the joint food-web model involves both lags and simultaneous interactions, i.e. trophic interactions occurring in the same year, two approaches involving randomized iterations were applied to account for these issues. After this step, the model was validated using data from the period 2008–2011. Details on both the set-up of the food-web model and its validation are described in the electronic supplementary material.

#### (iii) Simulation of scenarios

The validated food-web model was then used to assess the ecosystem regeneration potential under varying fishing pressure in combination with two sets of environmental conditions (figure 2, step 3). We defined a depletion–regeneration scenario where the cod fishing mortality (*F*) was gradually increased from 0 to a maximum of 1.4 (i.e. close to the historical maximum) and decreased again to 0 by applying a sequential change in the exploitation rate of *F* = 0.05. This scenario was run under two contrasting temperature and salinity conditions: (i) those found before 1989, which were favourable for cod, and (ii) those occurring after the regime shift, being less favourable for cod [18]. As the latter conditions still largely prevail, we will refer to these two scenarios as past (first regime) and current (second regime), respectively (figure 1*d*). For each simulation, temperature and salinity values were randomly sampled (with replacement) from the observations, corresponding to past and current regimes (see the electronic supplementary material, figure S5 and S6). All analyses were performed using R software v. 2.5.1 (www.r-project.org).

### (c) Bio-economic analysis

In order to assess the economic consequences of the depletion–regeneration scenario a bio-economic analysis was performed (figure 2, step 4). We estimated the profit of the commercial cod fishery, the CS and the annual RP for our scenarios of variable exploitation pressure under past and current temperature and salinity conditions. CS is a monetary quantification of consumer-related welfare. It is calculated by analysing the difference between what consumers are willing to pay for fish relative to its market price, and is mainly determined by harvest levels. RP quantifies the economic costs of increased variability in biomass for both profits and consumer welfare.

#### (i) Profit

We assume biomass growth dynamics for cod described by the following general equation:2.1
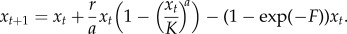


The biomass growth function contains as special cases the logistic function *rx*(*1 − x/K*) for *a* = *1* and the Fox [40] function *rx* ln(*K/x*) for *a* = *0* (which can be seen by taking the limit *a* → *0* and applying l'Hospital's rule). As we are interested in sustainable economic yield, we consider a dynamic equilibrium with *x_t+t_* = *x_t_* = *x*. Rearranging this formula, we received the following relationship between stock size and fishing mortality:2.2
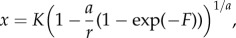
which we estimate by means of nonlinear ordinary least squares.

Further, we assume a profit function *pH − cF*, where *H* are cod landings, *p* is the market price for cod, and cost of effort, *cF*, is assumed to be proportional to instantaneous fishing mortality *F* with proportionality factor equal to marginal cost *c*. For the cost parameter we use the estimate from [41], which is *c* = 72.9 million € with a standard error of 19.8 million €. For the price, we assume an inverse demand function of the type *p*(*H*) = *p*_0_*H*^−η^. We use the estimate *η* = 0.23 from [42], and calibrate *p*_0_ = 559 € ton^−1^ of cod, such that the inverse demand function leads to a price of 1095 € ton^−1^ of cod at landings of 0.0538 million tons with price and landings data from [41]. See the electronic supplementary material for more information.

#### (ii) Consumer surplus and risk premium

Inverse demand is a measure for the consumer's willingness to pay for fish. With the downward-sloping inverse demand function of the type *p*(*H*) = *p*_0_*H*^−η^, the aggregate willingness to pay for fish exceeds the market value *p*(*H*)*H*. This gives rise to a CS of fish consumption, which is obtained as2.3
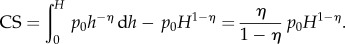


CS depends on harvest levels.

The annual RP additionally quantifies the costs of increased variability in biomass and associated harvest. We quantify RP for total economic welfare, i.e. the sum of profits and CS. As the cod price is sensitive to harvest levels [42], revenue is a concave function of harvest. Jensen's inequality implies that expected revenues are lower with a higher fluctuation of the harvest. Similarly, as CS is a concave function for harvest, the expected CS also decreases with harvest uncertainty. The RP associated with fluctuating harvest is defined as the difference between summed-up profits and CS at the expected biomass as compared with profits plus CS with fluctuating biomass. Higher variability gives rise to higher costs (see the electronic supplementary material).

All computations for the bio-economic module were done with Matlab (R2011A).

## 3. Results and discussion

### (a) Individual model fits

Our final food-web model consisted of cod as top predator, the two forage fish species herring and sprat, as well as the copepod *P. acuspes* and cladocerans. The latter entered the model only as covariate. The individual model fits show which, how and under what circumstances the different variables relate to each other (figure 3; electronic supplementary material, table S2–S5).

Our results show that cod is positively affected by the 2 year lagged biomass of *P. acuspes* (figure 3*a*), reflecting the beneficial feeding effect of this copepod on cod larvae survival and recruitment [43]. Furthermore, cod is negatively related to sprat, but only at intermediate to high biomass levels (figure 3*b*). Although sprat is an important prey for cod, the negative effect may reflect significant sprat predation on cod eggs [44], particularly at higher biomasses. Note that owing to pronounced model uncertainty at the extremes the weak-positive effect at maximum sprat biomass should be treated with caution. These two partial effects capture the two types of feedback mechanisms described in the ecosystem, the positive relationship of *P. acuspes* on cod (first regime) and the prey-to-predator loop (second regime). Finally, there is an obvious linear negative relationship to fishing pressure (figure 3*c*).

Sprat shows a nonlinear positive relationship to summer temperature (figure 3*d*), representing its positive effects on recruitment [45]. This effect is conspicuous up to about 4°C, above which increasing temperature does not lead to increased sprat biomass. Cod shows a linear negative effect on sprat (figure 3*f*), indicating its role as a top predator.

Finally, the negative relationship with cladocerans (figure 3*e*) denotes strong top-down control. Such negative effects of prey on predators are a frequent statistical result in top-down structured systems. For instance in the Black Sea, Llope *et al.* [39] found a negative effect of zooplankton on jellyfish for the regime when the latter were most abundant and, consequently, consumption was at its maximum. The same effect shifted to positive for the alternative regime (low abundance of jellyfish) when predation was less intense and the control turned to be bottom-up. These findings suggest that if predation is strong (runaway consumption, senso Strong [46]), the pattern displayed is that of a negative effect of the predator on the prey as it would only be possible to observe large numbers of prey when the abundance of its predator is low. An alternative model with the same covariates but excluding cladocerans would render the same shape for the temperature and cod partial effects and cause only a slight decrease in *r*^2^ (0.54 versus 0.6). Although cladocerans are not connected to any other model components and as such has little effect on model dynamics, it provides an understanding of food-web structure.

Herring and *P. acuspes* displayed non-additive dynamics depending on the biomass of cod. The threshold was lower for herring than for *P. acuspes*, 246 564 and 462 502 tons, respectively. Herring responds negatively to fishing (figure 3*g*) independently of the level of cod biomass. In addition, when cod biomass is low (and consequently sprat is high) competition with sprat becomes conspicuous as a negative effect of sprat biomass (figure 3*h*) [47]. *Pseudocalanus acuspes* is negatively impacted by small pelagics (herring and sprat) for the low cod biomass regime (figure 3*j*). Alternatively, above the cod threshold *P. acuspes* is positively related to salinity (figure 3*k*), probably owing to its positive effect on reproduction and maturation [48]. This result agrees with Casini *et al.* [17] showing that the dynamics of zooplankton is being driven either by hydrography or sprat predation depending on the level of cod.

The individual models together represent the general functioning of the system. Figure 3 summarizes those key linkages between components described above, which include some regime-dependent interactions defined by the level of cod in the ecosystem. When cod is abundant (more than 450 000 ton), its predation pressure on sprat releases zooplankton from top-down control. Consequently, *P. acuspes*, and possibly also cladocerans, increase in biomass and become regulated by environmental factors. This allows favourable bottom-up processes (e.g. high salinities) to propagate upwards, first via a positive effect on *P. acuspes*, which in turn, positively affect cod with a lag of 2 years. For the alternative regime (cod spawner biomass < 450 000 ton), small pelagics and particularly sprat control zooplankton.

### (b) Ecosystem regeneration pathways

The food-web model proved to reproduce the past dynamics reasonably well, as well as the recent increase in cod biomass (see the electronic supplementary material, figure S4).

**Figure 4.**
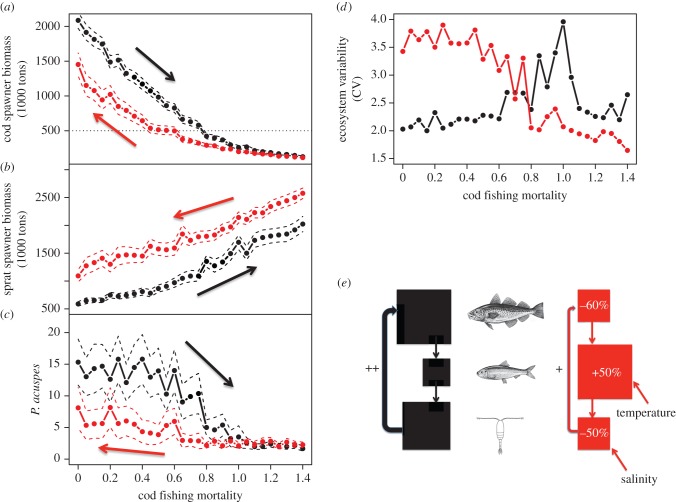
New ecosystem baseline with a lower stability. Response of cod (*a*), sprat (*b*) and *Pseudocalanus acuspes* (*c*) and the overall coefficient of ecosystem variability (*d*) to increased and subsequently decreased cod exploitation level (fishing mortality) under past (black) and current (red) conditions of temperature and salinity. Schematic of the variability in the strength of the prey-to-predator loop (including cod, top; sprat, middle and *P. acuspes*, bottom) leading to different regeneration pathways and lower baseline biomasses (indicated by sizes of squares as well as percentage changes) under past (black) and current (red) hydroclimatic conditions (*e*).

Our simulations show that regeneration pathways differ between past and current temperature and salinity conditions. Current environmental conditions result in biomasses that are lower for cod (figure 4*a*), higher for sprat (figure 4*b*) and lower for *P. acuspes* (figure 4*c*). Additionally, variability in biomass of the different food-web components increases in the current regime with decreasing cod fishing mortality, as illustrated by an increase in the coefficient of variation (CV) of simulated biomasses by up to 200% (figure 4*d*).

The difference in baseline and amount of variability can be explained by the climate's influence on the stabilizing feedback between cod and *P. acuspes* [18]. Generally, a reduction in cod exploitation causes an increase in cod biomass, a decrease in sprat owing to higher predation, and an increase in *P. acuspes* owing to lower sprat predation [49]. This so-called trophic cascade [50] is reinforced by a positive feedback (figure 4*e*), as a larger *P. acuspes* population will in turn positively affect cod larval recruitment and survival [43]. Forcing the model simulation with favourable temperature and salinity conditions (past regime) resulted in a strengthening of this feedback loop, maintained the system within the high cod regime, which in turn dampened the oscillations. Under same fishing mortality but current environmental conditions the feedback weakens and the prey-to-predator loop is favoured as higher temperatures enhance sprat recruitment [45] and lower salinities impair reproduction and maturation of *P. acuspes* [48]. Furthermore, *P. acuspes* currently experiences a larger salinity range (figure 1), which results in higher population variability. When the conditions open a window for this bottom-up effect to affect cod (with a lag of 2 years), this variability is also propagated to cod biomass.

Our simulation results support the existence of a feedback loop between sprat, *P. acuspe*s and cod [17,18] and demonstrates for the first time, to our knowledge, that multiple drivers synergistically affect the strength of the feedback loop under a range of exploitation rates and climate conditions. Current salinity and temperature conditions reduce the stabilizing effect of the feedback, leading to a weaker and more variable recovery pathway for cod. Hence, the Baltic Sea ecosystem probably cannot recover to its previous state, but instead regenerates towards a new, and more variable, ecosystem baseline.

It is worth noting that in our simulations we only focused on the synergistic effects between temperature, salinity and cod fishing mortality. Other stressors, e.g. sprat and herring fishing or eutrophication, would have probably affected the model structure and dynamics. In addition, we do not specifically account for any changes in life-history traits (size, rates) or in the spatial distributions of the species. We assume that such changes are at least partially reflected in the underlying data, e.g. biomass estimates, and therefore implicitly accounted for in the model set-up and simulations. Also, we consider the Gotland Basin as representative of the Central Baltic Sea for the lower trophic levels and hydrographical conditions. While acknowledging that this is a simplification of a complex system, we think the results provide new insight into the regeneration potential of the Baltic Sea.

### (c) Economic consequences

**Figure 5.**
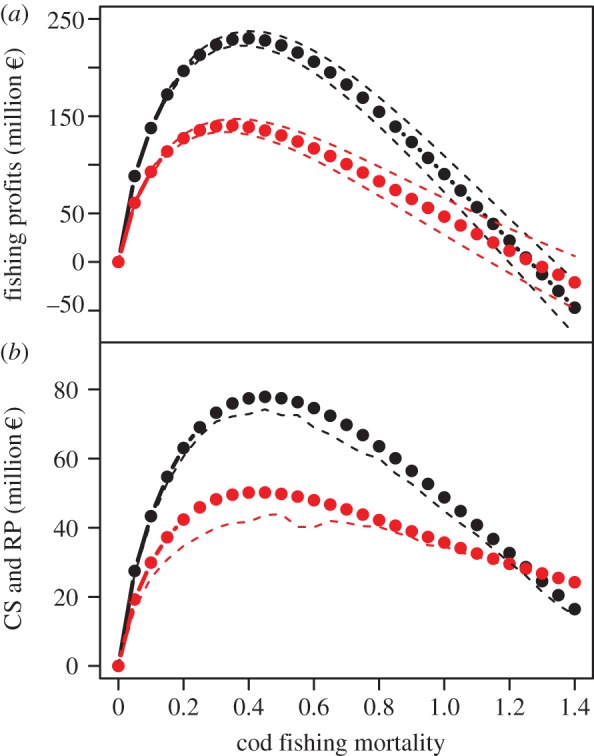
Economic profits and societal costs. Sustainable economic yields (*a*) and corresponding CS and RP (*b*) at different cod exploitation levels (fishing mortality) under past (black) and current (red) conditions of temperature and salinity. Dashed lines indicate 95% confidence limits (*a*) and CSs minus RPs (*b*).

The last step of our analysis focused on the evaluation of the direct and indirect economic implications of an altered productivity of cod, the most important species in this regard (figure 2). We found that while the economically optimal exploitation levels (aka *F*) differ only slightly (figure 5*a*) between the past and current temperature and salinity conditions, the annual profit is considerably lower (140 compared with 230 million €). Total economic costs have to also include the costs to society, in particular losses in CS. CS amounts to approximately 30% of fishing profits under optimal exploitation, and is, like direct fishing profits, considerably reduced under the current temperature and salinity conditions (shown as dots in figure 5*b*). In addition, the annual RP–a quantification of the costs of increased cod biomass variability—is higher under current conditions and amounts to another 6 million €, which have to be subtracted from welfare (the resulting reduced welfare levels are shown as dashed lines in figure 5*b*).

Overall, the change in ecosystem baseline is estimated to cause a total annual loss of approximately 123 million €, of which almost 30% are indirect costs. These results indicate that the economic baseline not only shifted in parallel to the ecological baseline, but that the current conditions may not be able to support as many viable fishing units as before, and imply a higher uncertainty for fishermen.

## 4. Conclusion

Climate change is projected to cause drastic increases in sea surface temperature of the world's oceans in general, and in the Baltic also lower salinities [51,52]. At the same time, efforts are being made worldwide to regenerate the ecosystems to favourable highly productive states [53]. In this study, we show that the pathway of ecosystem regeneration is, besides fishery management, conditionally dependent on the interaction of climate and human pressures and that the output of this interaction implies severe economic and societal costs. Our results show that the environmental conditions determine not only the level of achievable baselines but also—what is most important in socio-economic terms—their degree of variability.

We think that this study is relevant to managers and policy makers by providing a new perspective to the potential bio-economics of the Baltic Sea. Our results point out that: (i) the regeneration of an ecosystem to an economic target is not straightforward, as there are multiple interacting drivers involved that need be considered and understood; and (ii) an accurate evaluation of a given management strategy should account for these drivers and incorporate nonlinear and regime-dependent dynamics, as these features have been observed and determine the final outcome. It is important that the developing concept of adaptive resilience which should guide future policies embraces this evidence in order to maintain our ecosystems healthy, productive and sustainable for future generations [54].

## Supplementary Material

ELECTRONIC SUPPLEMENTARY MATERIAL for: Climate and fishing steer ecosystem regeneration to uncertain economic futures

## Data accessibility

All data are deposited in Dryad (manuscript Dryad ID: doi:10.5061/dryad.m1s8s).

## References

[1] Worm B 2009 Rebuilding global fisheries. Science 325, 578–585. (10.1126/science.1173146) 19644114

[2] Lotze HK Coll M Magera AM Ward-Paige C Airoldi L 2011 Recovery of marine animal populations and ecosystems. Trends Ecol. Evol. 26, 595–605. (10.1016/j.tree.2011.07.008) 21852017

[3] Jones HP Schmitz OJ 2009 Rapid recovery of damaged ecosystems. PLoS ONE 4, e0005653 (10.1371/journal.pone.0005653) PMC2680978 19471645

[4] Verdonschot PFM Spears BM Feld CK Brucet S Keizer-Vlek H Borja A Elliott M Kernan M Johnson RK 2012 A comparative review of recovery processes in rivers, lakes, estuarine and coastal waters. Hydrobiologia 704, 453–474. (10.1007/s10750-012-1294-7)

[5] Folke C 2006 Resilience: The emergence of a perspective for social–ecological systems analyses. Glob. Environ. Change 16, 253–267. (10.1016/j.gloenvcha.2006.04.002)

[6] Campbell LM Gray NJ Hazen EL Shackeroff JM 2009 Beyond baselines: rethinking priorities for ocean conservation. Ecol. Soc. 14 (online).

[7] Duarte CM Conley DJ Carstensen J Sánchez-Camacho M 2009 Return to Neverland: shifting baselines affect eutrophication restoration targets. Estuar. Coasts 32, 29–36. (10.1007/s12237-008-9111-2)

[8] Harris JA Hobbs RJ Higgs E Aronson J 2006 Ecological restoration and global climate change. Rest. Ecol. 14, 170–176. (10.1111/j.1526-100X.2006.00136.x)

[9] Perry RI Cury P Brander K Jennings S Möllmann C Planque B 2010 Sensitivity of marine systems to climate and fishing: concepts, issues and management responses. J. Mar. Syst. 79, 427–435. (10.1016/j.jmarsys.2008.12.017)

[10] Scheffer M Carpenter S Foley JA Folke C Walker B 2001 Catastrophic shifts in ecosystems. Nature 413, 591–596. (10.1038/35098000) 11595939

[11] Scheffer M Carpenter SR 2003 Catastrophic regime shifts in ecosystems: linking theory to observation. Trends Ecol. Evol. 18, 648–656. (10.1016/j.tree.2003.09.002)

[12] Conversi A 2014 A holistic view of marine regime shifts that spans multiple ecosystems and stressors. Phil. Trans. R. Soc. B 370, 1–8. (10.1098/rstb.2013.0279)

[13] Nyström M 2012 Confronting feedbacks of degraded marine ecosystems. Ecosystems 15, 695–710. (10.1007/s10021-012-9530-6)

[14] Daskalov GM 2002 Overfishing drives atrophic cascade in the Black Sea. Mar. Ecol. Prog. Ser. 225, 53–63.

[15] Conversi A Umani SF Peluso T Molinero JC Santojanni A Edwards M 2010 The Mediterranean sea regime shift at the end of the 1980s, and intriguing parallelisms with other European basins. PLoS ONE 5, e10633 (10.1371/journal.pone.0010633) 20502704 PMC2873283

[16] Hare SR Mantua NJ 2000 Empirical evidence for North Pacific regime shifts in 1977 and 1989. Prog. Oceanogr. 47, 103–145.

[17] Casini M Hjelm J Molinero JC Lovgren J Cardinale M Bartolino V Belgrano A Kornilovs G 2009 Trophic cascades promote threshold-like shifts in pelagic marine ecosystems. Proc. Natl Acad. Sci. USA 106, 197–202. (10.1073/pnas.0806649105) 19109431 PMC2629246

[18] Möllmann C Diekmann R Muller-Karulis B Kornilovs G Plikshs M Axe P 2009 Reorganization of a large marine ecosystem due to atmospheric and anthropogenic pressure: a discontinuous regime shift in the Central Baltic Sea. Glob. Change Biol. 15, 1377–1393. (10.1111/j.1365-2486.2008.01814.x)

[19] EC. 2008 Council Regulation No. 1342/2008 of 18 December 2008 establishing a long-term plan for cod stocks and the fisheries exploiting those stocks and repealing. Brussels, Belgium: EC.

[20] Eero M Koster FW Vinther M 2012 Why is the Eastern Baltic cod recovering? Mar. Policy 36, 235–240. (10.1016/j.marpol.2011.05.010)

[21] Cardinale M Svedang H 2011 The beauty of simplicity in science: Baltic cod stock improves rapidly in a ‘cod hostile’ ecosystem state. Mar. Ecol. Prog. Ser. 425, 297–301. (10.3354/meps09098)

[22] Möllmann C Blenckner T Casini M Gårdmark A Lindegren M 2011 Beauty is in the eye of the beholder: management of Baltic cod stock requires an ecosystem approach. Mar. Ecol. Prog. Ser. 431, 293–297. (10.3354/meps09205)

[23] Diekmann R Möllmann C 2010 Integrated ecosystem assessments of seven Baltic Sea areas covering the last three decades. In ICES cooperative research report, p. 92 Copenhagen, Denmark: ICES.

[24] Harvey CJ Cox SP Essington TE Hansson S Kitchell JF 2003 An ecosystem model of food web and fisheries interactions in the Baltic Sea. ICES J. Mar. Sci. 60, 939–950. (10.1016/s1054-3139(03)00098-5)

[25] Lindegren M Mollmann C Nielsen A Stenseth NC 2009 Preventing the collapse of the Baltic cod stock through an ecosystem-based management approach. Proc. Natl Acad. Sci. USA 106, 14 722–14 727. (10.1073/pnas.0906620106) PMC2732840 19706557

[26] Tomczak MT Niiranen S Hjerne O Blenckner T 2012 Ecosystem flow dynamics in the Baltic Proper: using a multi-trophic dataset as a basis for food–web modelling. Ecol. Mod. 230, 123–147. (10.1016/j.ecolmodel.2011.12.014)

[27] Ciannelli L Chan KS Bailey KM Stenseth NC 2004 Nonadditive effects of the environment on the survival of a large marine fish population. Ecology 85, 3418–3427. (10.1890/03-0755)

[28] ICES. 2013 *Report of the ICES/HELCOM Working Group on Integrated Assessments of the Baltic Sea (WGIAB)*, p. 40 Copenhagen, Denmark: ICES.

[29] Köster FW 2003 Recruitment of Baltic cod and sprat stocks: identification of critical life stages and incorporation of environmental variability into stock-recruitment relationships. Sci. Mar. 67, 129–154.

[30] ICES. 2012 *Report of the Baltic Fisheries Assessment Working Group (WGBFAS)*, p. 692 Copenhagen, Denmark: ICES.

[31] Möllmann C Kornilovs G Sidrevics L 2000 Long-term dynamics of main mesozooplankton species in the Central Baltic Sea. J. Plan. Res. 22, 2015–2038.

[32] Otto S Diekmann R Flinkman J Kornilovs G Möllmann C 2014 Habitat heterogeneity determines climate impact on zooplankton community structure and dynamics. PLoS ONE 9, e90875 (10.1371/journal.pone.0090875) 24614110 PMC3948703

[33] MacKenzie BR Hinrichsen H-H Plikshs M Wieland K Zezera AS 2000 Quantifying environmental heterogeneity: habitat size necessary for successful development of cod (*Gadus morhua)* eggs in the Baltic Sea. Mar. Ecol. Prog. Ser. 193, 143–156.

[34] Lehmann A Krauss W Hinrichsen HH 2002 Effects of remote and local atmospheric forcing on circulation and upwelling in the Baltic Sea. Tellus Ser. A 54, 299–316. (10.1034/j.1600-0870.2002.00289.x)

[35] Rodionov SN 2004 A sequential algorithm for testing climate regime shifts. Geophys. Res. Lett. 31, 1–4. (10.1029/2004gl019448)

[36] Andersen T Carstensen J Hernández-García E Duarte CM 2009 Ecological thresholds and regime shifts: approaches to identification. Trends Ecol. Evol. 24, 49–57. 18952317 10.1016/j.tree.2008.07.014

[37] Hastie T Tibshirani R 1990 Generalized additive models. New York, NY: Chapman & Hall. 10.1177/096228029500400302 8548102

[38] Wood S 2006 Generalized additive models. An introduction with R, vol 1 Boca Raton, FL: Chapman & Hall.

[39] Llope M Daskalov GM Rouyer TA Mihneva V Chan K-S Grishin AN Stenseth NC 2011 Overfishing of top predators eroded the resilience of the Black Sea system regardless of the climate and anthropogenic conditions. Glob. Change Biol. 17, 1251–1265. (10.1111/j.1365-2486.2010.02331.x)

[40] Fox WW 1970 An exponential surplus-yield model for optimizing exploited fish populations. Trans. Am. Fish. Soc. 99, 80 (10.1577/1548-8659(1970)99<80:aesmfo>2.0.co;2)

[41] Quaas MF Requate T Ruckes K Skonhoft A Vestergaard N Voss R 2013 Incentives for optimal management of age-structured fish populations. Res. Energy Econ. 35, 113–134. (10.1016/j.reseneeco.2012.12.004)

[42] Nielsen M 2006 Trade liberalisation, resource sustainability and welfare: the case of East Baltic cod. Ecol. Econ. 58, 650–664. (10.1016/j.ecolecon.2005.08.013)

[43] Hinrichsen HH Möllmann C Voss R Köster FW Kornilovs G 2002 Biophysical modeling of larval Baltic cod (*Gadus morhu*a) growth and survival. Can. J. Fish. Aquat. Sci. 59, 1858–1873. (10.1139/f02-149)

[44] Köster FW Möllmann C 2000 Trophodynamic control by clupeid predators on recruitment success in Baltic cod? ICES J. Mar. Sci. 57, 310 (10.1006/jmsc.1999.0528)

[45] MacKenzie BR Köster FW 2004 Fish production and climate: sprat in the Baltic Sea. Ecology 85, 784–794.

[46] Strong DR 1992 Are trophic cascades all wet? Differentiation and donner-control in speciose ecosystem. Ecology 73, 747–754. (10.2307/1940154)

[47] Casini M Bartolino V Molinero JC Kornilovs G 2010 Linking fisheries, trophic interactions and climate: threshold dynamics drive herring (*Clupea harengus*) growth in the Central Baltic Sea. Mar. Ecol. Prog. Ser. 413, 241–252. (10.3354/meps08592)

[48] Renz J Hirche HJ 2006 Life cycle of *Pseudocalanus acuspe*s Giesbrecht (Copepoda, Calanoida) in the Central Baltic Sea. I. Seasonal and spatial distribution. Mar. Biol. 148, 567–580. (10.1007/s00227-005-0103-5)

[49] Möllmann C Müller-Karulis B Kornilovs G St John MA 2008 Effects of climate and overfishing on zooplankton dynamics and ecosystem structure: regime shifts, trophic cascade, and feedback coops in a simple ecosystem. ICES J. Mar. Sci. 65, 302–310. (10.1093/icesjms/fsm197)

[50] Casini M Lovgren J Hjelm J Cardinale M Molinero JC Kornilovs G 2008 Multi-level trophic cascades in a heavily exploited open marine ecosystem. Proc. R. Soc. B 275, 1793–1801. (10.1098/rspb.2007.1752) PMC2587786 18460432

[51] Hoegh-Guldberg O Bruno JF 2010 The impact of climate change on the world's marine ecosystems. Science 328, 1523–1528. (10.1126/science.1189930) 20558709

[52] Meier HEM 2012 Comparing reconstructed past variations and future projections of the Baltic Sea ecosystem: first results from multi-model ensemble simulations. Environ. Res. Let. 7, 034005 (10.1088/1748-9326/7/3/034005)

[53] Costanza R 1997 The value of the world's ecosystem services and natural capital. Nature 387, 253–260. (10.1038/387253a0)

[54] Suding KN Gross KL Houseman GR 2004 Alternative states and positive feedbacks in restoration ecology. Trends Ecol. Evol. 19, 46–53. (10.1016/j.tree.2003.10.005) 16701225

