# Supplementary material for: Climate and fishing steer ecosystem regeneration to uncertain economic futures
Source: Proc Biol Sci. 2015 Mar 22;282(1803):20142809. doi: 10.1098/rspb.2014.2809 (PMC4345453; doi:10.1098/rspb.2014.2809)
Supplement: ELECTRONIC SUPPLEMENTARY MATERIAL for: Climate and fishing steer ecosystem regeneration to uncertain economic futures [file rspb20142809supp1.pdf]

ELECTRONIC SUPPLEMENTARY MATERIAL for:

Climate and fishing steer ecosystem regeneration to uncertain economic futures

Thorsten Blenckner[1], Marcos Llope, Christian Möllmann, Rudi Voss, Martin F. Quaas, Michele Casini, Martin Lindegren, Carl Folke, Nils Chr. Stenseth

[1] *Corresponding author: Thorsten Blenckner, Stockholm Resilience Centre, Stockholm University, Kräftriket 2B, 106 91 Stockholm, Sweden, +46 (0) 86747669, Thorsten.blenckner@su.se*

Contents

1. Model setup and individual model selection procedures
    - 1.1 General model characteristics
    - 1.2 Individual model selection
    - 1.3 Model assumptions
2. Food web model
    - 2.1 Linkage of individual models
    - 2.2 Simulations
    - 2.3 Validation
    - 2.4 Scenarios
3. Economic analysis
4. Tables
5. Supplementary figures
6. References

1. Model setup and individual model selection procedures

1.1 General model characteristics

For the selection of the individual models we used non parametric regressions in two fashions:

(1) The common Generalized Additive Models (GAM) [1,2] in the form of:

$$Y_t = \propto + \sum f_i(X_{it}) + \varepsilon_t \qquad \text{(eq. 1)}$$

where Y_t is the response variable (i.e., biomass of a given trophic level in year t), α is the intercept and X_{it} are predictor covariates in year t (or year t-1 if lags apply), which relate to Y_t through the corresponding smooth functions f_i. ε_t is the random noise term assumed to be normally distributed with zero mean and finite variance.

(2) Its non-additive (threshold) formulation (tGAM):

$$Y_t = \propto + \begin{cases} \sum f_{1i}(X_{it}) \; if \; codSSB \leq \phi \\ \sum f_{2i}(X_{it}) \; if \; codSSB > \phi \end{cases} + \varepsilon_t \qquad \text{(eq. 2)}$$

where, as for simple GAM, α is a single intercept (i.e., one constant over the whole period) and ε_t the random noise. The difference here is that a given predictor (X_{it}) is allowed to switch from function f_{1i} to function f_{2i} in its relation to the response variable Y_t depending on a level Φ of the threshold variable (cod spawning stock biomass) [1, 2].

1.2 Individual model selection

Model selection was performed as follows. First, an individual GAM was estimated for all fish and zooplankton species, as well as for chlorophyll a, following standard model

selection procedures (see Material and Methods in main text). We could only find significant and robust models for cod, sprat, herring, and *P. acuspes* (Fig. 3).

In some cases, time lags were chosen: (1) for the cod model a time lag of two years for *P. acuspes* was most significant and explained more than 30% of the variance. We are confident that a 2 year lag is mechanistically plausible as the cod age classes 2 and 3 make up about 50-60% of its total biomass [3]. Hence, this lag would represent predation of cod larvae on *P. acuspes*. (2) The same approach was used with sprat. As the assessment data come from the very beginning of each year [4], temperature and *P. acuspes* was lagged by one year to best match sprat biomass.

To compare non additive models (tGAMs) with their corresponding fully additive (GAM) formulations it is necessary to account for the additional parameter used for the threshold search [3]. The Generalized Cross Validation (GCV) used for the GAM model selection is only a (good) approximation of the real CV and it does not take into account the fact that a grid search has been put in place to find the value of the threshold. Thus, we used the genuine Cross Validation (gCV) to compare between the two model alternatives. gCV equals the average squared leave-one-out prediction errors; the leave-one-out prediction is obtained by removing one data case at a time from the model fitting and predicting its value from the resulting model.

1.3 Model assumptions

The underlying statistical assumptions of all models were tested as follows. Temporal independence was verified by plotting the autocorrelation function (ACF), the normality of residuals was assessed by plotting theoretical versus sample quintiles (Q-Q plot). The homogeneity of variance was evaluated by plotting residuals over time. The residuals of

the models were tested to be uncorrelated over time and to follow a normal and homoscedastic distribution (Fig. S1).

2. Food web model, simulations and validation

2.1 Linkage of individual models

The selected 'optimal' individual models were linked together in a joint food web model. This is done by allowing the predicted biomass from a given individual model (e.g., *P. acuspes*) to feed into those models where this trophic level has an effect (e.g., cod model). By doing this, we let the food web interact according to the empirically estimated relationships with the external conditions (environmental data and fishing pressure) as solely independent drivers (see [5] for details).

Simulating the observed time series using the combined set of models was not straightforward as there were (1) lags involved and (2) sprat and cod affect each other simultaneously (in the same year). To circumvent these two difficulties we did the following:

(1) Lags. *P. acuspes* in the cod model is lagged by two years. It is not possible to simulate the first two observations for cod (years 1974 and 1975) using this model configuration. To get round this issue, we first created a seed of values. Specifically, we randomly drew a year within the first regime (1974-1987) and predicted the biomass of *P. acuspes* using its corresponding model and the conditions during that year. By repeating the process twice we got a set of values for *P. acuspes* that we assumed to be two realizations corresponding to the first regime. These values for *P. acuspes* were subsequently used to feed the cod model two steps (years) ahead.

(2) Iterations. As cod and sprat depend on each other in the same year a number of 20 iterations were used to get the final values. The process is simple and consists of (i) feed cod with the predicted value for sprat and next (ii) feed sprat using the outcome of the cod model until the final values converge. Convergence happened after a maximum of 9-10 iterations. The figure S2 gives two realizations of this process, corresponding to years 1981 and 2005. In 1981, cod progressively increased whereas sprat decreased (Fig. S2a) until they converged close to the observed value that year (Fig. S2b). In the second example, the cod-sprat trajectory departed from the first regime space to fall onto the second regime (Fig. S2c).

2.2 Simulations

To evaluate the performance of the food web model we simulated the observed time series with covariates fixed at their observed values. The simulations are therefore only based on the dynamic structure of the system. Noise was added to the biotic variables by sampling (with replacement) the model residuals. To preserve the contemporaneous correlation of errors, a whole vector of errors for the four trophic levels corresponding to a randomly sampled year was used at a time. One thousand Monte Carlo simulations were run for each trophic level from which the mean and the 95% prediction bands were calculated.

The goal of this simulation was to be able to reproduce the two regimes described in the Baltic, which are characterized by high cod/low sprat and low cod/high sprat, respectively. These two phases can be easily tracked in a cod vs. sprat trajectory plot. Figure S3 shows these trajectories for both the observations and simulations. Although the simulations span a narrower range, the two regimes can be easily identified.

2.3 Validation

We validated the performance of our model by showing that it reproduces well both past and recent Baltic food web dynamics, and especially the recent increase in cod (Fig. S4). The overestimation of cod predicted by our model for years 2006-2009 might be due to the fact that some other factors limit the cod recovery but illustrate that our model results do not underestimate the effects of hydroclimate and cod recovery.

2.4 Scenarios

The two hydroclimate scenarios (past and current) were constructed by resampling individual variables (with replacement) from the series of observations, before and after year 1988, respectively. Figure S5 shows the actual observations from which the variables were randomly drawn and Figure S6 shows the fishing pressure gradient and the associated past and current scenario used to feed the models. Apart from using the actual observations to construct the scenarios, as explained before, we also tried creating new series from scratch based on the variance and autocorrelation of the observed series in order to test any possible memory effect in the environment. Figure S7 show the scenarios produced in this way. The results were the same.

3. Economic analysis

The fitted cod biomass growth function is displayed both under past and current hydroclimate conditions (Fig. S8 and Fig. S9)

Under the past conditions we obtain the following profit estimates:

$$r_1 = 0.663 \text{ with 95\% confidence interval } [0.633, 0.692]$$
$$K_1 = 2.124 \text{ with 95\% confidence interval } [2.086, 2.162]$$
$$a_1 = 0.831 \text{ with 95\% confidence interval } [0.772, 0.889]$$

For the second regime with current hydroclimate conditions, we obtain

$$r_2 = 0.517 \text{ with 95\% confidence interval } [0.461, 0.573]$$
$$K_2 = 1.365 \text{ with 95\% confidence interval } [1.313, 1.416]$$
$$a_2 = 0.486 \text{ with 95\% confidence interval } [0.350, 0.622]$$

Equilibrium profits at a fishing mortality F are

$$p_0 K^{1-\eta}\left(1-exp\left(-F\right)\right)^{1-\eta}\left(1-\frac{a}{r}\left(1-exp\left(-F\right)\right)\right)^{(1-\eta)/a} - c\,F \qquad \text{(eq. 3)}$$

For a global sensitivity analysis, we draw 1000 random parameter sets, assuming normally distributed parameter values for r_i, K_i, a_i and c_i with means given by the point estimates and variances as determined by the variance-covariance matrix obtained from the nonlinear OLS regression for the biological parameters and determined by the square of the standard error for the cost parameter c. For each parameter set we compute equilibrium profits at given fishing mortality levels. The standard deviation of the resulting set of profit values is the computation error reported in Figure 5 in the main text. To determine the error for the optimal fishing mortality level, we determine the optimal fishing mortality for each of the 1000 parameter sets and use the standard deviation of the resulting set of optimal fishing mortalities as the standard error.

The risk premium is calculated as follows:

$$
\begin{aligned}
RP = {} & E\left[p_0\left(\left(1-exp\left(-F\right)\right)x\right)^{1-\eta} - c\,F + \frac{\eta}{1-\eta}\,p_0\left(\left(1-exp\left(-F\right)\right)x\right)^{1-\eta} \right] \\
& - p_0\left(\left(1-exp\left(-F\right)\right)E\left[x\right]\right)^{1-\eta} - \frac{\eta}{1-\eta}\,p_0\left(\left(1-exp\left(-F\right)\right)E\left[x\right]\right)^{1-\eta} + c\,F \\
= {} & E\left[\frac{p_0}{1-\eta}\left(\left(1-exp\left(-F\right)\right)x\right)^{1-\eta} \right] - \frac{p_0}{1-\eta}\left(\left(1-exp\left(-F\right)\right)E\left[x\right]\right)^{1-\eta}
\end{aligned}
$$

(eq. 4)

Where *E[]* denotes the expectation with respect to environmental fluctuations. We assumed normal distributions for biomass, using the expected biomasses at the different fishing mortality levels and the variance of biomasses obtained from the biological simulations.

4. Tables

Table S1 Data table describing the variables used for the modelling. SD = ICES sub-divisions, GOR=Gulf of Riga. LATFRA= Latvian Fish Resource Agency, BED=Baltic Environmental database, HCO=Helmholtz Centre for Ocean research, Kiel, Germany

Variable	Unit	Area	Time	Years	Source	Reference
Cod total biomass	Tonnes	SD 25-32	Annual	1974-2009	ICES	www.ices.dk
Cod fishing mortality	Age 4-7	SD 25-32	Annual	1974-2009	ICES	www.ices.dk
Sprat spawner biomass	Tonnes	SD 22-32	Annual	1974-2009	ICES	www.ices.dk
Sprat fishing mortality	Age 3-5	SD 22-32	Annual	1974-2009	ICES	www.ices.dk
Herring spawner biomass	Tonnes	SD 25-29, 32 excl GOR	Annual	1974-2009	ICES	www.ices.dk
Herring fishing mortality	Age 3-5	SD 25-29, 32 excl GOR	Annual	1974-2009	ICES	www.ices.dk
Acartia spp.	mg m^3	Gotland Basin	May, August	1974-2009	LATFRA	[6] (updated)
Temora longicornis	mg m^3	Gotland Basin	May, August	1974-2009	LATFRA	[6] (updated)
Pseudocalanus acuspes	mg m^3	Gotland Basin	May, August	1974-2009	LATFRA	[6] (updated)
Cladocerans	mg m^3	Gotland Basin	August	1974-2009	LATFRA	[6] (updated)
Chlorophyll *a*	mg m^3	Gotland Basin	April-May June-Sept.	1979-2009	ICES	[7] (updated)
Sea surface temperature	Degree Celcius	Gotland Basin	May, July	1974-2013	BED	Nest.su.se
Mid-water temp. (40-60 m)	Degree Celcius	Gotland Basin	May, July	1974-2009	BED	Nest.su.se
Mid-water sal. (80-100 m)	Psu	Gotland Basin	May	1974-2009	BED	Nest.su.se
Cod reproductive volume	Km^2	Central Baltic	Annual	1974-2009	HCO	[8] (updated)
Baltic Sea Index		Central Baltic	Winter	1974-2009	HCO	[9] (updated)

Table S2 Cod model results with the covariates cod fishing mortality (F), sprat and *P. acuspes*. Intercept, effective degrees of freedom (edf) and significance (p-value) of the covariates, and R-squared (R^2) for the cod model (see Fig. 3 in the main text).

	**estimate**	**p-value**
intercept	5.53	<0.001
threshold	none	
**covariate**	**edf**	**p-value**
F	1.00	<0.001
Sprat	2.72	<0.001
P. acuspes	1.00	<0.001
R^2 (adj) = 0.742		
Deviance explained: 78.1%		

Table S2 Sprat model results with the covariates temperature (Temp), biomass of cladocerans (Clad) and cod. Intercept, effective degrees of freedom (edf) and significance (p-value) of the covariates, and R-squared (R^2) for the sprat model (see Fig. 3 in the main text).

	**estimate**	**p-value**
intercept	5.17	<0.001
threshold	None	
**covariate**	**edf**	p-**value**
Temp.	1.98	0.022
Clad	1.00	0.024
Cod	1.00	0.001
R^2 (adj) = 0.600		
Deviance explained: 65%		

Table S3 Herring model results with the covariates sprat if cod is low (regime 1) and the fishing mortality of herring (FHer). Intercept, effective degrees of freedom (edf) and significance (p-value) of the covariates, and R-squared (R^2) for the herring model (see Fig. 3 in the main text).

		**estimate**	**p-value**
	intercept	6.05	<0.001
	threshold (θ)	5.39	
**regime**	**covariate**	**edf**	**p-value**
Cod≤ θ	Sprat	1.98	<0.001
-	FHer	1.77	<0.001
	R^2 (adj) = 0.853		
	Deviance explained: 86.9%		

Table S4 *Pseudocalanus acuspes* model results with the covariates of planktivorous fish (Plan, herring plus sprat when cod biomass is low (regime 2) and salinity (Sal) when the cod is high (regime 1). Intercept, effective degrees of freedom (edf) and significance (p-value) of the covariates, and R-squared (R^2) for the *Pseudocalanus* model (see Fig. 3 in the main text).

		**estimate**	**p-value**
	intercept	3.05	<0.001
	threshold (θ)	5.66	
**regime**	**covariate**	**edf**	**p-value**
Cod≤ θ	Plan	1.00	0.046
Cod>θ	Sal	1.91	<0.001
	R^2 (adj) = 0.43		
	Deviance explained: 48%		

5. Supplementary Figures

Figure S1. Prediction performance and assessment of residuals for the individual models of cod (COD), sprat (SPR), herring (HER) and *Pseudocalanus acuspes* (PSE). The first column shows time series of predictions vs. observations. The following four columns show the inspection of residuals for the assumptions of independence (autocorrelation function), normality (Q-Q plot), and homoscedasticity (residuals vs. time).

Figure S2. Iteration process. Left hand panels (a & c) show the biomasses for cod and sprat over 20 iterations (open circles) for years 1981 and 2005, respectively. Right hand panels (b & d) show the corresponding cod-sprat trajectories. The numbered labels track the iteration number. The final value, chosen at iteration number = 20 is represented by a filled circle while the observed value is shown in red.

Figure S3. Observations vs simulations. Phase space cod/sprat trajectories for the observed (black dots) and simulated (blue dots) values.

Figure S4. Validation exercise. Predictions of new observations of cod biomass (in log tonnes) using the cod individual model (Table 1), which was fitted on the calibration period (1974-2005) and are run now until 2009.

Figure S5. Time series of the covariates selected by the models. (a) summer sea surface temperature (TMP), (b) abundance of cladocerans (CLA), (c) spring mid-water salinity

(SLN), (d) fishing effort on cod (F cod) and (e) fishing effort on herring (F her). All but the fishing pressure on cod were used to construct the two scenarios. The horizontal line in 1988 divide the two hydroclimate regimes corresponding to past (fill circles) and current (open circle) from which values were sampled.

Figure S6. Fishing gradient (a) and environmental scenario obtained by randomly resampling the observations: (b) temperature, (c) salinity, (d) cladocerans, and (e) fishing pressure on herring. The values for the past conditions are shown in black while grey is used for the current hydroclimate.

Figure S7. Fishing gradient (a) and environmental scenario obtained by generating the time series based on the structure of the observations: (b) temperature, (c) salinity, (d) cladocerans, and (e) fishing pressure on herring. The values for the past conditions are shown in black while grey is used for the current hydroclimate.

Figure S8. Cod biomass in dependency of fishing mortality under past environmental conditions. The fitted cod biomass growth function is displayed.

Figure S9. Cod biomass in dependency of fishing mortality under current environmental conditions. The fitted cod biomass growth function is displayed.

Figure S1

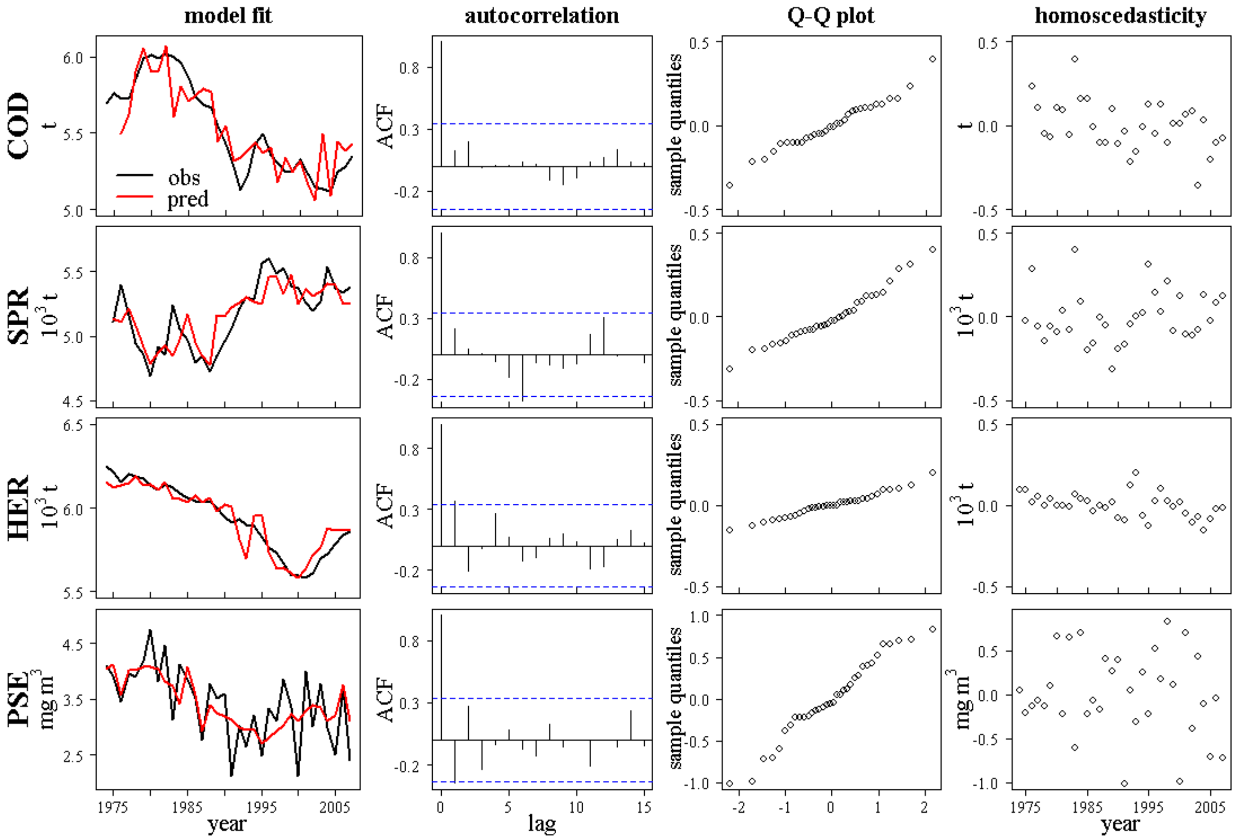
model fit
autocorrelation
Q-Q plot
homoscedasticity
COD
SPR
HER
PSE
obs
pred
ACF
sample quantiles
year
lag

Figure S2

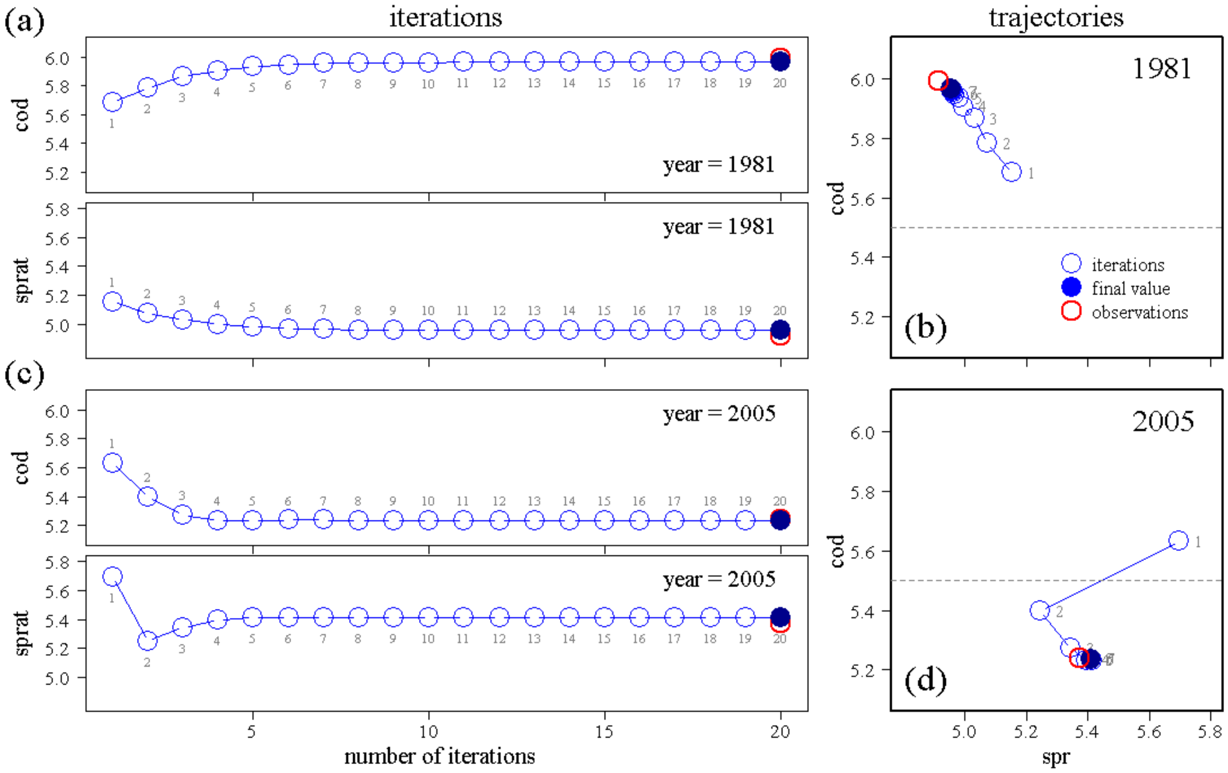

(a)
iterations
trajectories
cod
sprat
year = 1981
year = 2005
(b)
1981
iterations
final value
observations
(c)
(d)
2005
number of iterations
spr

Figure S3

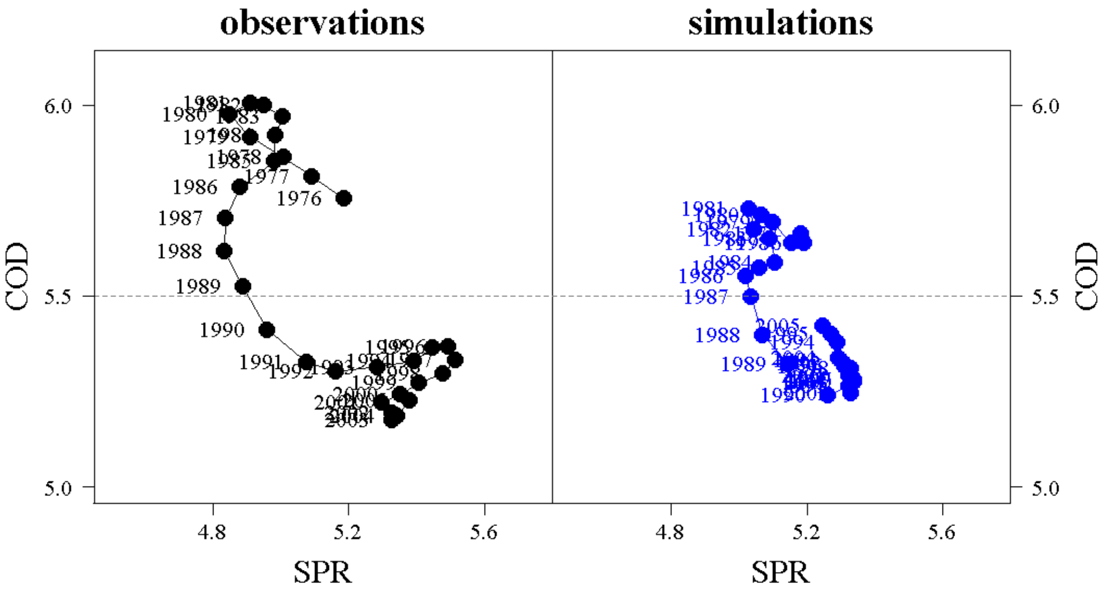
observations
simulations
COD
COD
SPR
SPR
6.0
5.5
5.0
4.8
5.2
5.6
1976
1977
1986
1987
1988
1989
1990
1988
1989
1987

Figure S4

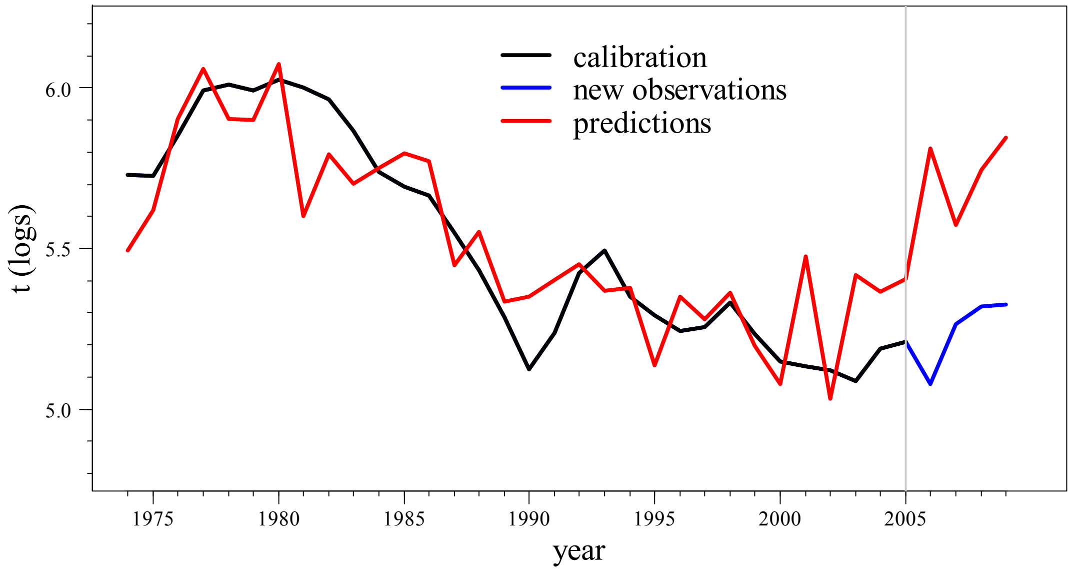
calibration
new observations
predictions
6.0
5.5
5.0
t (logs)
1975
1980
1985
1990
1995
2000
2005
year

Figure S5

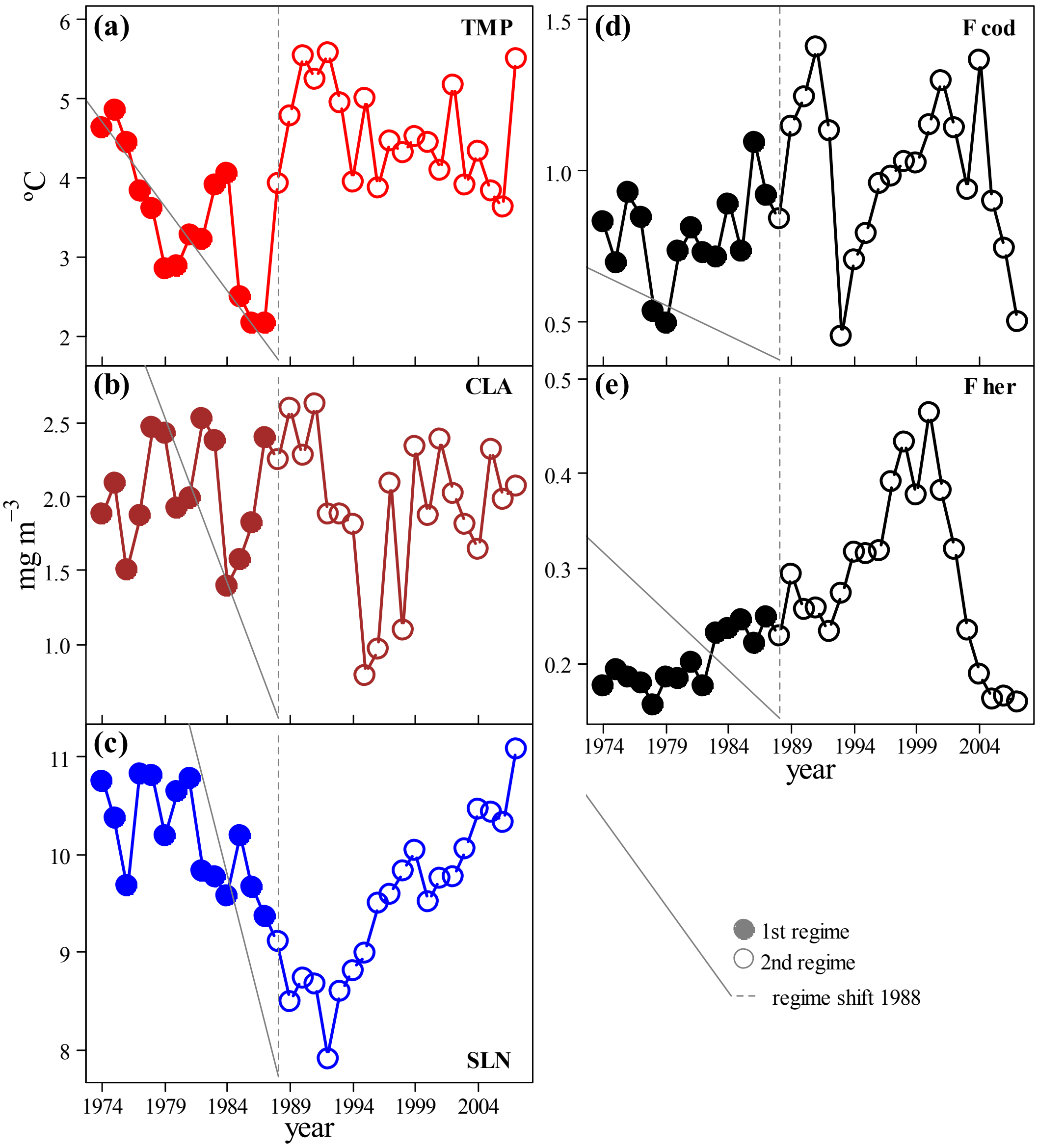
(a)
TMP
°C
(b)
CLA
mg m−3
(c)
SLN
(d)
F cod
(e)
F her
1974 1979 1984 1989 1994 1999 2004
year
1st regime
2nd regime
regime shift 1988

Figure S6

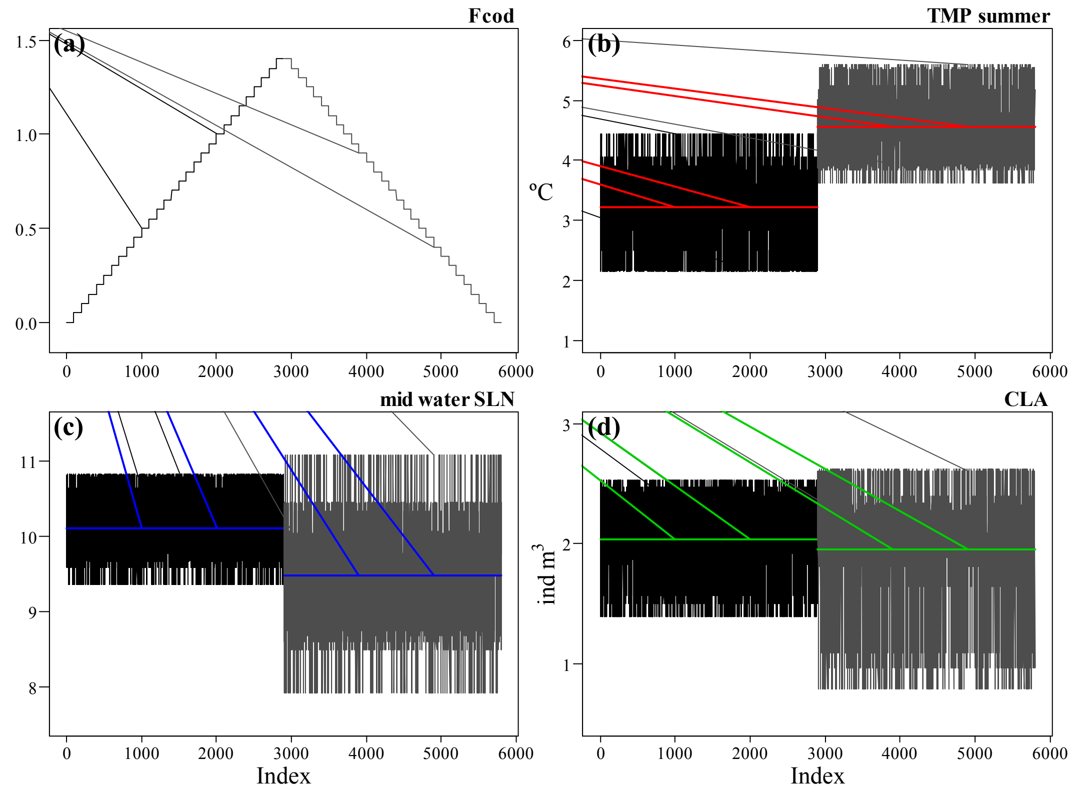

Figure S6

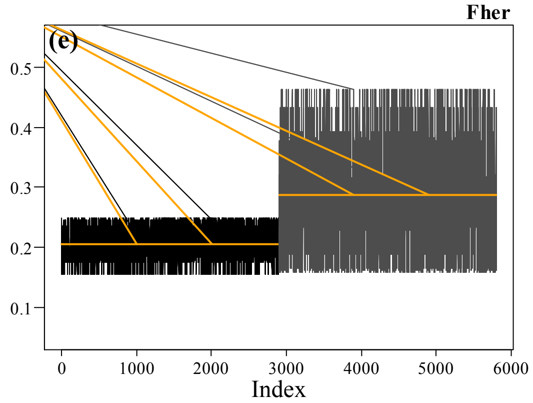

Figure S7

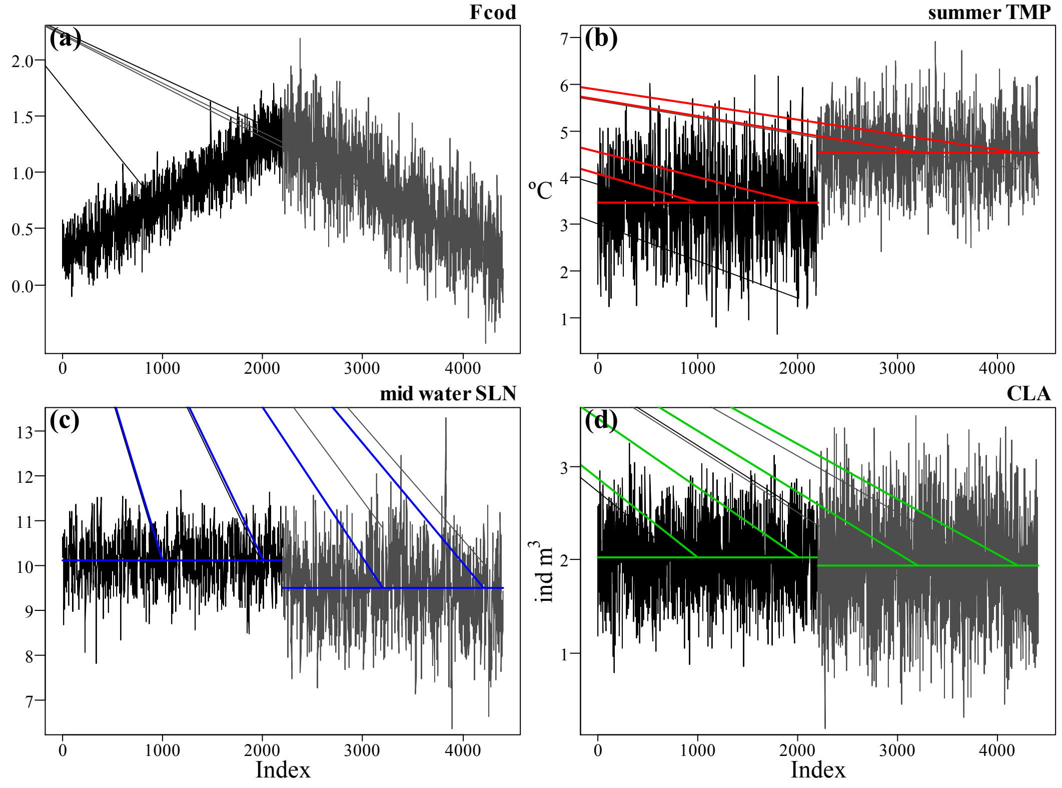

Fcod
(a)
2.0
1.5
1.0
0.5
0.0
0
1000
2000
3000
4000
summer TMP
(b)
7
6
5
4
3
2
1
°C
0
1000
2000
3000
4000
mid water SLN
(c)
13
12
11
10
9
8
7
0
1000
2000
3000
4000
Index
CLA
(d)
3
2
1
ind m3
0
1000
2000
3000
4000
Index

Figure S7

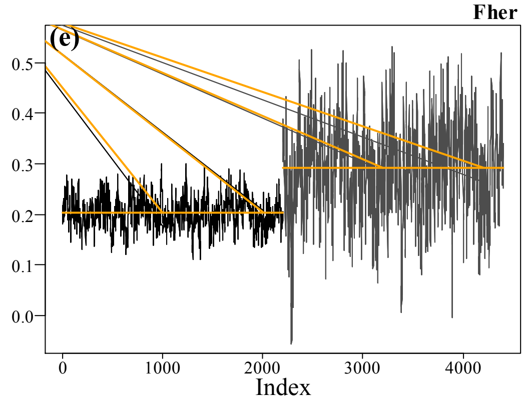

Figure S8

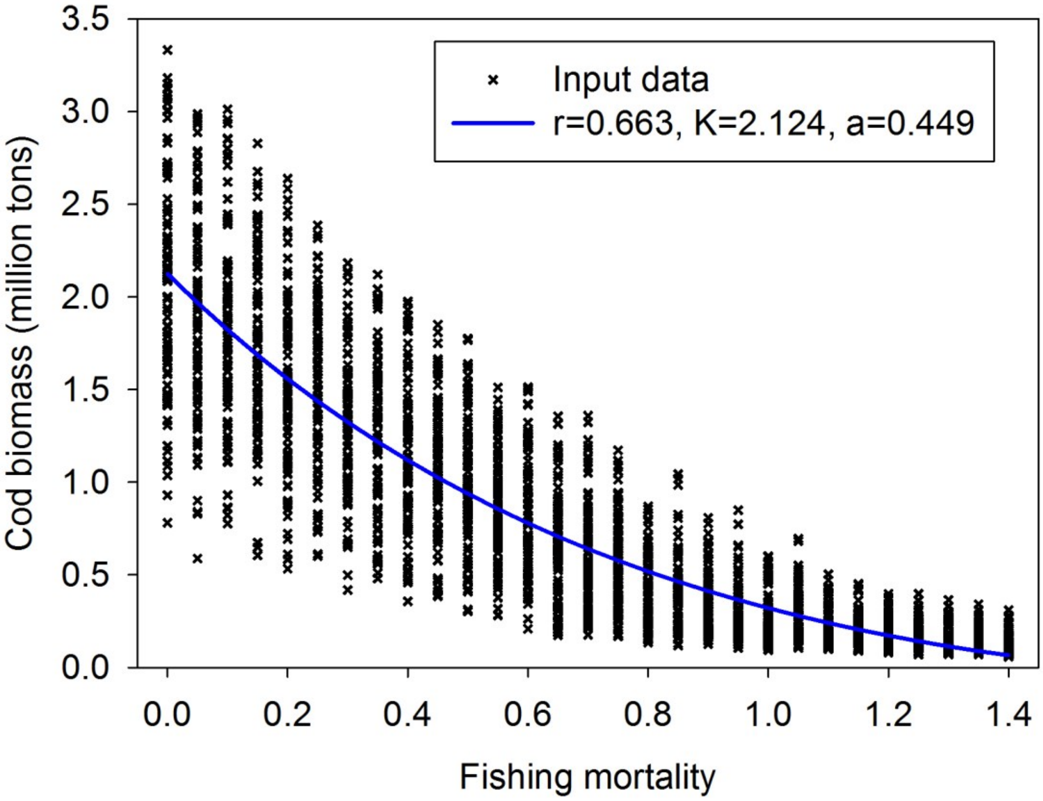
Input data
r=0.663, K=2.124, a=0.449
Cod biomass (million tons)
Fishing mortality
3.5
3.0
2.5
2.0
1.5
1.0
0.5
0.0
0.0
0.2
0.4
0.6
0.8
1.0
1.2
1.4

Figure S9

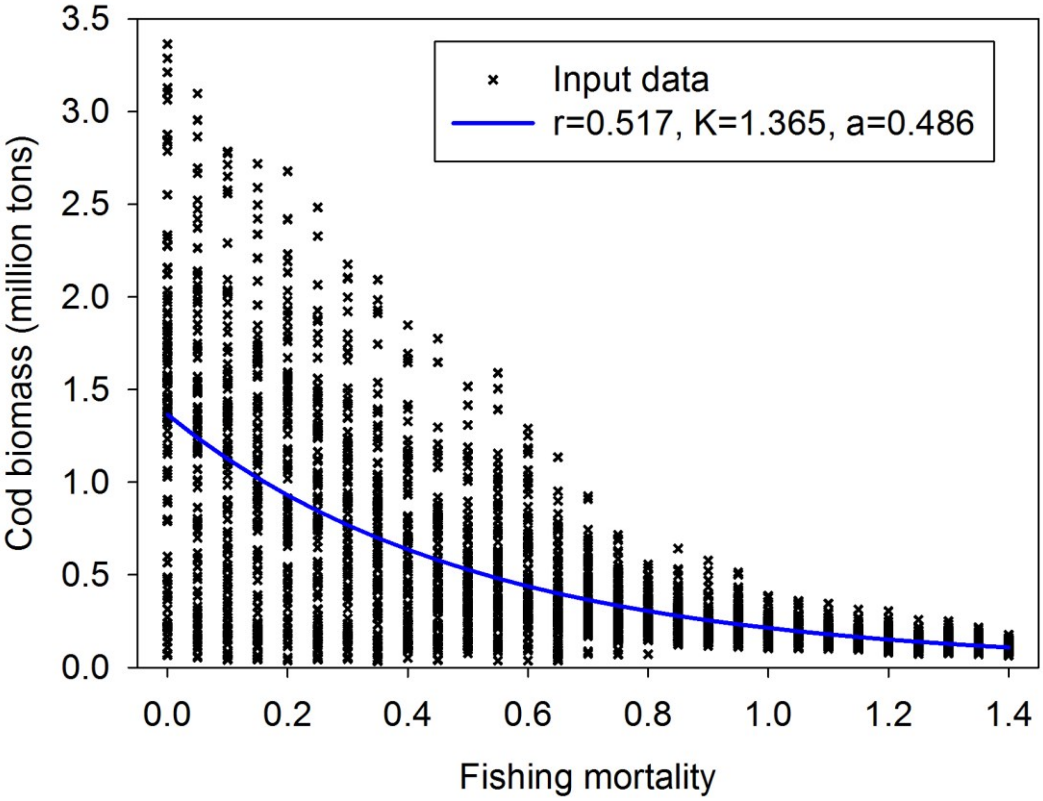
Input data
r=0.517, K=1.365, a=0.486
Cod biomass (million tons)
3.5
3.0
2.5
2.0
1.5
1.0
0.5
0.0
0.0
0.2
0.4
0.6
0.8
1.0
1.2
1.4
Fishing mortality

6. References

1. Wood S. 2006 *Generalized Additive Models. An Introduction with R, Vol 1*. Boca Raton, FL, USA, Chapman & Hall.
2. Hastie T., Tibshirani R. 1990 *Generalized Additive Models*. New York, Chapman & Hall.
3. Ciannelli L., Chan K.S., Bailey K.M., Stenseth N.C. 2004 Nonadditive effects of the environment on the survival of a large marine fish population. *Ecology* **85**(12), 3418-3427. (doi:10.1890/03-0755).
4. ICES. 2012 Report of the Baltic Fisheries Assessment Working Group (WGBFAS). (p. 692 Copenhagen, ICES.
5. Llope M., Daskalov G.M., Rouyer T.A., Mihneva V., Chan K.-S., Grishin A.N., Stenseth N.C. 2011 Overfishing of top predators eroded the resilience of the Black Sea system regardless of the climate and anthropogenic conditions. *Global Change Biology* **17**(3), 1251-1265. (doi:10.1111/j.1365-2486.2010.02331.x).
6. Möllmann C., Kornilovs G., Sidrevics L. 2000 Long-term dynamics of main mesozooplankton species in the central Baltic Sea. *Journal of Plankton Research* **22**(11), 2015-2038.
7. Wasmund N., Uhlig S. 2003 Phytoplankton trends in the Baltic Sea. *Ices Journal of Marine Science* **60**(2), 177-186. (doi:10.1016/s1054-3139(02)00280-1).
8. MacKenzie B.R., Hinrichsen H.-H., Plikshs M., Wieland K., Zezera A.S. 2000 Quantifying environmental heterogeneity: habitat size necessary for successful development of cod (*Gadus morhua)* eggs in the Baltic Sea. *Mar Ecol Prog Ser* **193**, 143-156.
9. Lehmann A., Krauss W., Hinrichsen H.H. 2002 Effects of remote and local atmospheric forcing on circulation and upwelling in the Baltic Sea. *Tellus Series a-Dynamic Meteorology and Oceanography* **54**(3), 299-316. (doi:10.1034/j.1600-0870.2002.00289.x).
